# Practical Model for Energy Consumption Analysis of Omnidirectional Mobile Robot

**DOI:** 10.3390/s21051800

**Published:** 2021-03-05

**Authors:** Linfei Hou, Fengyu Zhou, Kiwan Kim, Liang Zhang

**Affiliations:** 1 School of Control Science and Engineering, Shandong University, Jinan 250061, China; 201600800091@mail.sdu.edu.cn (L.H.); zhoufengyu_sdu@163.com (F.Z.); 2 Department of Electrical & Electronics Engineering, Chungnam State University, Cheongyang 33303, Korea; kkw@cnsu.ac.kr; 3 School of Mechanical, Electrical and Information Engineering, Shandong University, Weihai 264209, China

**Keywords:** Mecanum mobile robots, minimum-energy control, energy modeling, energy measurements, complex environment

## Abstract

The four-wheeled Mecanum robot is widely used in various industries due to its maneuverability and strong load capacity, which is suitable for performing precise transportation tasks in a narrow environment. While the Mecanum wheel robot has mobility, it also consumes more energy than ordinary robots. The power consumed by the Mecanum wheel mobile robot varies enormously depending on their operating regimes and environments. Therefore, only knowing the working environment of the robot and the accurate power consumption model can we accurately predict the power consumption of the robot. In order to increase the applicable scenarios of energy consumption modeling for Mecanum wheel robots and improve the accuracy of energy consumption modeling, this paper focuses on various factors that affect the energy consumption of the Mecanum wheel robot, such as motor temperature, terrain, the center of gravity position, etc. The model is derived from the kinematic and kinetic model combined with electrical engineering and energy flow principles. The model has been simulated in MATLAB and experimentally validated with the four-wheeled Mecanum robot platform in our lab. Experimental results show that the accuracy of the model reached 95%. The results of energy consumption modeling can help robots save energy by helping them to perform rational path planning and task planning.

## 1. Introduction

The Mecanum wheel is the most common omnidirectional wheel structure used in practical applications. Compared with other omnidirectional wheel structures, it has high complexity capabilities [1]. Mecanum wheel robots have the advantage when it comes to stability, high load capacity, simple structure, and flexible movement. Therefore, they are widely used in industrial production [2]. While Mecanum wheel robots have the advantage of movement flexibility, they also consume more energy than ordinary wheeled robots [3].

In recent years, Mecanum wheel robots have been widely used in warehousing and logistics. With the increasing degree of automation of mobile robots, mobile robot energy models are becoming more complex. However, the energy modeling of Mecanum wheel mobile robots in complex environments has not been given enough attention. In this paper, we mainly study the power consumption modeling of the Mecanum wheel robot in upslope and downslope environments. A separate analysis of the effects of various factors on power consumption is presented, and a power consumption model in a complex environment of Mecanum wheel robots is proposed.

**Figure 1:**
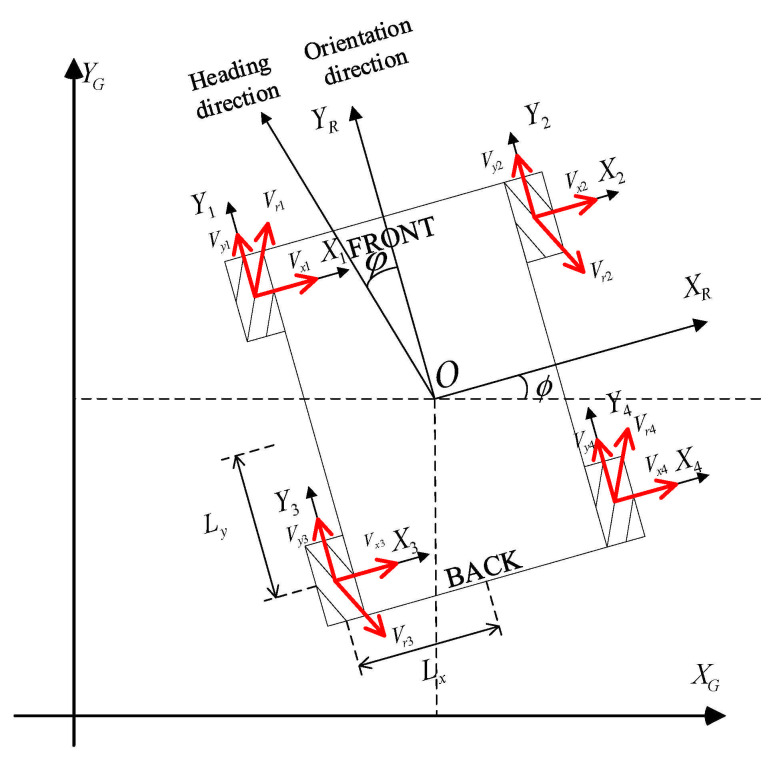
Schematic of the four-wheeled Mecanum wheel robot (FMWR).

The special structure of the four-wheeled Mecanum wheel robot (FMWR) (see Figure 1) gives it the ability to move omnidirectionally and with high loads [4]. The particular structure of the Mecanum wheel provides the robot with better flexibility than ordinary wheeled robots. The omnidirectional movement performance enables mobile robots to carry out better transportation tasks in crowded, narrow, or highly dynamic environments. However, the special structure and movement of the Mecanum wheel also increases the energy consumption of the robot, so, compared to ordinary wheeled robots, the Mecanum wheel robot has more energy consumption. [5]. Almost all robots need the energy from the batteries they carry [6]. The energy situation of the battery dramatically limits the task completion of the robots [7]. Energy-efficient strategies for mobile robots can expand the range of uses, perform more missions, and accomplish more complex operations [8,9,10]. Energy modeling has significance for battery energy management and range estimation of mobile robots [11,12,13]. Accuracy of energy models can help robots with task planning, energy prediction [14,15,16,17,18], sensor setup [19,20], and energy optimal path planning [21,22,23], and have very significant applications in autonomous planning, autonomous exploration [24], and even robot design.

At present, the main research on the energy consumption of Mecanum wheel robots focuses on the energy consumption of the smooth-running phase of the Mecanum wheel robot during the motion on the plane. The energy modeling for this part has been relatively well developed [25,26]. However, there is a lack of analysis of the difference in energy consumption of the Mecanum wheel robot between different terrains, and there is no modeling analysis of the factors affecting the energy consumption of the robot, the energy consumption fluctuations in the transition phase between the two stable operation phases are also only briefly explained [25,26]. This results in incomplete modeling scenes, and the model cannot adapt to the various environments in which the robot operates. Moreover, the accuracy of the modeling needs to be improved.

The main contribution of this paper is to analyze and model the energy consumption of a Mecanum wheel robot in complex environments, the analysis of the factors that have a significant impact on the robot’s energy consumption, which are represented as parameters in the modeling. Finally, the difference between the power consumption of Mecanum wheel robots and ordinary wheeled robots due to their special structure is emphasized. According to the previous study, we divided the energy consumption of the Mecanum wheel robot into three major systems (control system, motion system, and sensing system), modeled the energy consumption of each of these three systems, and analyzed the interactions and connections between the energy consumption of the three systems. In this paper, we focus on the energy consumption fluctuations of robots, which are often neglected in previous energy consumption studies. We investigate the energy consumption of the Mecanum wheeled robot during uphill and downhill slopes, analyze the differences between the Mecanum wheel robot and ordinary wheeled robots during its motion due to its special structure, and study the fluctuations of energy consumption during the transition phase of its motion on different surfaces and the reasons for them.

The main objective of the study is to increase the applicability of the Mecanum wheel robot modeling as well as to improve the accuracy of the modeling to help the Mecanum wheel robot predict its energy consumption more accurately. By modeling the energy consumption of the robot in complex terrain and adding the analysis of the factors affecting the energy consumption of the robot, the scenarios for modeling the energy consumption of the Mecanum wheel can be further increased. The analysis of the factors affecting the energy consumption can help to improve the accuracy of the modeling effectively. The modeling results can help the robot find the path with the lowest energy consumption during path planning and reduce the robot’s energy consumption, as well as help the robot predict the energy required for the task and perform reasonable task planning.

## 2. Related Works

At present, there are many known studies on power consumption models of omnidirectional mobile robots, including omnidirectional wheels and Mecanum wheels robots, terrain and trajectory planning, and so on. Sedat Dogru and Lino Marques [1] modeled the energy consumption of the skid steer robot throughout its motion and analyzed each of the factors affecting the robot’s energy consumption individually. B. K. Kim et al. [8,27,28,29] focused on the optimal energy consumption trajectory planning strategy for a three-wheeled omnidirectional wheel robot, proposed that the energy consumption of the robot could be reduced by reducing the speed change of the robot, and planned the lowest energy consumption path for the three-wheeled omnidirectional wheel robot by kinematic and dynamic modeling, combined with the Pontryagin minimax principle, which was verified to save about 30% energy consumption than the ordinary path planning algorithm. Xie [30] investigated the trajectory planning method for the minimum energy consumption of the Mecanum wheel robot. The method used was to apply the established energy consumption model of the Mecanum wheel to the trajectory planner based on the extended dynamic window method. According to the verification, it can meet the purpose of reducing energy consumption while achieving the online obstacle avoidance function. However, in the study of modeling energy consumption of the Mecanum wheel robot, only the energy consumption of the motion system was modeled, neglecting the energy consumption of the control system and sensing system.

Researches are also evolving rapidly in numerous specific scenarios. Amir Sadrpour et al. [31] examined the problem of mission prediction and battery energy prediction for unmanned ground-operated vehicles using batteries as energy sources that undertook specific tasks. Jesús Morales et al. [32] modeled the energy consumption of a skid steer robot on the hard ground through the two perspectives of power generated by rigid terrain and power provided by the motor. Broderick et al. [19] found that, for small and lightweight robots, the energy consumption of the robot’s non-motor systems (e.g., sensing, communication, and computing) accounted for a large percentage of the robot’s energy consumption. Therefore, appropriate scheduling strategies, as well as advanced energy conservation techniques and energy-efficient materials, could have a major effect on reducing the energy consumption of robots [33]. Structures, such as wheeled robots with redundant brakes [34], limb robots [35], and snake robots [36] that can replace the energy consumption of the motion system are also being investigated. Motor resistances have been identified as the main source of power dissipation in the traction system of wheeled robots, which can be minimized with an appropriate velocity profile [27]. Brateman et al. [20] pointed out that energy consumption could be effectively reduced by coordinating the relationship between the robot motor speed and the processing frequency of the processor and the scanning frequency of the sensors. Power demanded by one motor is almost independent of the speed commanded to the other, whereas in skid-steer, the power required by one motor heavily depends on the speed of the other [37].

## 3. Energy Modeling

In order to improve the accuracy of robot modeling in complex environments, we divide the power consumption of robots into three major systems, namely motion system, control system, and sensing system—modeling and analyzing the energy consumption of these three systems separately. Finally, the connection between the power consumption of the three systems is sought.

### 3.1. Motion System

The first is that the power consumption of the motion system conforms to the following formula [1].

(1) $$E_{motion}=\int P_{motor}dt=\int I_{a}E_{m}dt$$

$E_{motion}$ is the energy consumed by the robot’s motion system, $P_{motor}$ is the total power of the robotic motion system, $I_{a}$ is the total current flowing through the robot’s motion system. $E_{m}$ is the electromotive force on the robot motor.

The power consumption of the robot’s motion system is the power consumption of the robot’s DC motor, but it is complicated to determine the measurement of the current for the robot’s motor, because the currents around the motor are not the same, and the current of the robot motor is not the same at different times. Thus, it is very inaccurate to measure the power consumption of the robot motor in this way. Therefore, we decompose the power consumption of the motor and divide the power consumption of the motor into consumption at the place.

(2) $$P_{motor}=P_{colo}+P_{lolo}+P_{outpu}{}_{t}$$

$P_{motor}$ is the power of the robot’s motor, $P_{colo}$ is the power of the robot’s copper consumption, $P_{lolo}$ is the power consumption of the robot’s iron loss, $P_{outpu}{}_{t}$ is the mechanical output of the robot.

(3) $$P_{colo}=I_{a}{}^{2}R_{a}$$

$P_{colo}$ is the power of the motor to lose energy when the robot is in motion, $I_{a}$ is the current flowing through the motor, $R_{a}$ Is the internal resistance of the robot motor.

For a DC motor, the torque T and the rotation speed of the shaft $\omega_{shaft}$ are proportional to the armature current $I_{a}$ and the electromotive force E. At the same time, the torque and speed output of the motor are proportional to the force $F_{wheels}$ and the angular velocity *w* of the wheel. τ is the conversion factor of force and energy consumption, which is related to the radius of the wheel and the connection of the wheel to the motor. $k\left(T\right)$ is the speed energy factor of the robot, β(T) is the temperature energy consumption coefficient of the robot; their values, as well as the correspondence with speed and temperature, are given in Equations (30) and (31) below.

(4) $$P_{colo}=\tau \cdot F_{wheel}^{2}+k\left(T\right)+\beta (T)*t$$

(5) $$P_{lolo}=P_{edlo}+P_{hylo}+P_{other}$$

The iron loss of the robot was divided into three parts, where $P_{edlo}$ is eddy current loss, $P_{hylo}$ is hysteresis loss, $P_{other}$ is the iron loss after removing eddy current loss and hysteresis loss of other parts of the loss.

Because the eddy current loss and hysteresis loss of the robot cannot be measured, the iron loss of the robot is directly related to the speed, acceleration, and running time of the robot.

(6) $$P_{lolo}=\lambda_{1}\sum_{i=1}^{4}\left(v_{i}*a\right)+\lambda_{2}\left(t*a_{i}*v^{2}\right)+\lambda_{3}\left(t^{2}*a*v^{2}\right)$$

$\lambda_{1}$, $\lambda_{2}$, and $\lambda_{3}$ are the energy consumption coefficients of the robot motors, $v_{i}$ is the real-time speed of each wheel of the robot, v is the actual motion speed of the robot, $a_{i}$ is the real-time acceleration corresponding to each wheel of the robot. According to the characteristics of the Mecanum wheel robot, the mechanical output of the robot can be subdivided into the mechanical energy of the output and the power consumption due to the friction losses caused by the characteristic structure of the Mecanum wheels, which is:

(7) $$P_{output}=P_{mechanical}+P_{fica}$$

(8) $$P_{mechanical}=I_{a}E_{b}$$

Through the principles of motor science, we can learn that the motor’s mechanical output is transformed into the torque T and rotational speed $\omega_{shaft}$ of the motor shaft, and they are proportional to the input current $I_{a}$ and input voltage $E_{b}$ of the motor. For the robot, the output force $F_{wheels}$ of the wheels is proportional to the torque T of the motor, while the rotational speed of the wheel is equal to the product of the torque $\omega_{shaft}$ of the motor and the diameter of the wheel [2].

(9) $$P_{mechanical}=k_{1}F_{wheels}{}^{2}+k_{2}\omega F_{wheels}$$

where $k_{1}$ and $k_{2}$ are proportionality constants, which represent information about the motor’s torque, armature resistance, and other parameters [2].

(10) $$P_{fica}=\mu_{1}\sum_{i=1}^{4}\left(N_{i}\cdot v_{i}\right)\cos\theta +\mu_{2}N\cdot v\cos\theta \cos\gamma$$

where $\mu_{1}$ and $\mu_{2}$ are the coefficients of friction of the robot wheels, their values and variation laws are given in Equations (25) and (26). $N_{i}$ is the positive pressure acting on each drive wheel, θ is the angle between the direction of robot motion and the positive direction of the robot, γ is the angle between the inclined plane and the horizontal plane of the robot movement

So:

(11) $$P_{output}=k_{1}F_{wheels}{}^{2}+k_{2}\omega F_{wheels}+\mu_{1}\sum_{i=1}^{4}\left(N_{i}\cdot v_{i}\right)\cos\theta +\mu_{2}N\cdot v\cos\theta \cos\gamma$$

So, the robotic motion system can be analyzed as:

(12) $$\begin{array}{ll} P_{motion} & =\lambda_{1}\sum_{i=1}^{4}\left(v_{i}*\frac{△v}{△t}\right)+\lambda_{2}\left(\sum_{i=1}^{4}\frac{△v_{i}}{△t}*v^{2}*t\right)+\lambda_{3}\left(t^{2}*\frac{△v}{△t}*v^{2}\right)+k_{1}F_{wheels}{}^{2}+k_{2}\omega F_{wheels}\\ & +\mu_{1}\sum_{i=1}^{4}\left(N_{i}\cdot v_{i}\right)\cos\theta +\mu_{2}N\cdot v\cos\theta \cos\gamma +\tau \cdot F_{wheels}{}^{2}+k\left(T\right)+\beta (T)*t \end{array}$$

The impact on the power consumption of the motion system in different environments will be analyzed below.

### 3.2. Control System

The control system mainly sends commands to the sensing system to control the sensors and poll the sensor readings periodically and sends commands to the motion system to control the motion state of each motor. It was found that the speed of the robot, the scanning frequency of the sensors and the running time of the robot all have an impact on the energy consumption of the control system. According to the different states of the robot operation, we can divide the power consumption of the robot control system into three phases: the standby phase, the phase in which the robot speed changes drastically, and the final smooth-running phase. The consumption of the control system of the robot in this test is mainly for processing the data of each sensor of the robot, analyzing the motion state of the robot, and controlling the motion state of the robot to perform the motion, according to the controller’s setting. Finally, the control system has the basic operating power consumption during the running process and the heat energy consumption generated by the increase in time during the running process [24].

(13) $$E_{control}=\left\{\begin{array}{l} E_{stanby}=\int P_{stanby}dt\\ E_{spech}=\int \left(\sum_{i=1}^{n}\rho_{i}\cdot fs_{i}\cdot \frac{v_{i}}{v_{\max}}+\rho_{1}\left(\sum_{i=1}^{4}\left|\frac{\Delta v_{i}}{\Delta t}\right|\right)+\rho_{2}\cdot t^{\frac{1}{10}}+P_{stanby}\right)\\ E_{stable}=\int \left(\sum_{i=1}^{n}\rho_{i}\cdot fs_{i}+\rho_{2}\cdot t^{\frac{1}{10}}+P_{stanby}\right)dt \end{array}\right.dt$$

$E_{control}$ is the power consumed by the robot control system during the motion, $E_{stanby}$ is the power consumption consumed by the robot control system in the standby state, $E_{spech}$ is the power consumption of the robot’s control system when the motion state of the robot changes, $E_{stable}$ is the power consumption of the robot’s control system during smooth operation.

$P_{stanby}$ is the power consumption of the motion system of the robot in the standby state. This part constitutes the basic power consumption of the robot control system. This part of the power consumption exists in all phases of robot operation. ρ is a parameter that characterizes the amount of data of the sensor of the robot, which is proportional to the amount of data generated by the robot’s sensor for data acquisition. For the control system, the more data generated by the robot’s sensing system, the more computing resources that need to be used, and the more energy the control system consumes. fs is the sampling frequency set by the robot’s sensor standard. For the robot of this experiment, the standard sampling frequency of each sensor has been set before the experiment was performed. In order to save power during motion and minimize power consumption, we combine the real-time sampling frequency of the robot with the speed of the robot. The sampling frequency of the robot’s sensing system is proportional to the real-time speed of the robot.

In addition, $\rho_{1}$ is the acceleration parameter of the robot, which characterizes the relationship between the power consumption and the acceleration of the control system during the speed change of the robot. $\sum_{i=1}^{4}\left|\frac{\Delta v_{i}}{\Delta t}\right|$ is the sum of the absolute values of the four drive wheel accelerations of the robot, because the control system can individually control each drive wheel of the robot during the movement.

$\rho_{2}$ is the time power consumption parameter of the control system. During the movement of the robot, the control system of the robot will dissipate part of the energy consumption in the form of heat energy due to the increase of the running time. This part of the power consumption can be expressed as a function of time.

### 3.3. Sensor System

The main function of the robot’s sensing system is to detect the environment around the robot and send the detected data to the robot’s control system for processing. The power consumption of the robot sensing system satisfies the following formula:

(14) $$E_{sensor}={\sum_{i=1}^{n}\left(P_{sensor}\right)}_{i}$$

$E_{sensor}$ represents the power consumption of the robot sensing system, ${\left(P_{sensor}\right)}_{i}$ is the power of the ith sensor, the power consumption of the sensing system can be superimposed on the sum of the power consumption of each sensor.

(15) $$P_{s}\left(f_{S}\right)=C_{S0}+C_{S1}\cdot f_{S}$$

The power consumption of a sensor is determined by both the type of sensor and the acquisition frequency. The energy consumption of different types of sensors varies greatly. For example, for cameras and LIDAR, the energy consumption varies greatly, and for the same sensor with different frequencies of data acquisition, the energy consumption also varies greatly, we can use a linear function to characterize the energy consumption of the sensor, where $C_{S0}$ represents the basic energy consumption of the sensor, and $C_{S1}$ represents the sensor constant coefficient of the sensor, their values only depend on the type of sensor [36].

## 4. The Influence of Various Factors on the Power Consumption of the Robot

Next, we will analyze in detail the various influencing factors affecting FMWR power consumption in a complex environment in detail:

### 4.1. Research on the Power Consumption of Robots Uphill and Downhill

#### 4.1.1. Power Analysis of Robot Uphill Process

The upslope of the robot mainly affects the speed and acceleration of the robot, which has a huge impact on the robot’s control system, motion system, and sensing system. The influence on the control system and the sensing system is mainly reflected in the change of the corresponding part of the robot’s power consumption due to the change of the speed; the speed change of the robot can be obtained by substituting it into the formula.

The following is mainly to analyze the power consumption of the motion system of the robot motion system during the ascending process. After analyzing the robot’s driving force rise and speed change caused by the angle problem during the uphill process, the impact on the iron loss is reflected in the speed. We do not conduct detailed analysis, and analyze the changes in iron loss and mechanical output.

**Figure 2:**
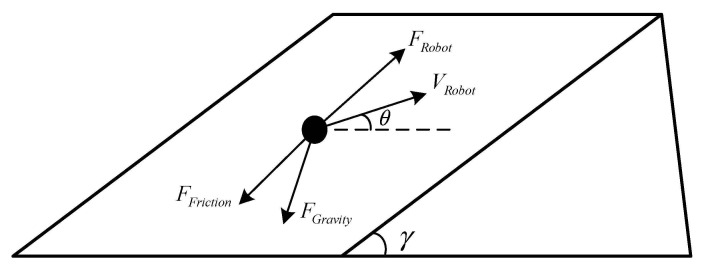
Schematic diagram of the force analysis along the slope direction during the uphill movement of the robot.

Figure 2 shows the analysis of the forces on the robot in the direction of the incline during the uphill movement. We simplified the force analysis of the robot during the motion, and only analyzed the force along the slope, which can help us simplify the force analysis and kinematic and dynamics analysis. Firstly, according to the force analysis (see Figure 2), the robot is subjected to several forces in the direction of the slope during its uphill movement. In addition to the driving force provided by the robot itself, the forces on the robot in the direction of the incline mainly include the component of the robot’s gravity in the direction of the incline and the frictional force acting on the robot. Because the robot is moving uphill, the velocity with respect to the slope is upward along the slope, so the frictional force acting on the robot is downward along the slope. Therefore, the direction of the combined force formed by the component of gravity along the slope and the friction force is downward along the slope. The robot needs to provide the upward force along the slope to ensure the balance of forces if it wants to maintain a stable motion on the slope. The drive force $F_{up}$ provided by the robot is upward along the incline, and the value of $F_{up}$ is the sum of the value of the component of gravity $F_{gravity}$ along the slope and the value of the friction force $F_{friction}$, i.e., $F_{up}=F_{gravity}+F_{friction}$. Because of the special construction of the Mecanum wheel, it is subjected to frictional forces, including both rolling friction between the wheel and the inclined surface and the sliding friction between the roller and the slope.

After stress analysis, it is found that the following equation exists during the ascending process:

(16) $$F_{up}=F_{gravity}+F_{friction}$$

(17) $$F_{gravity}=mg\sin\theta \sin\gamma$$

(18) $$F_{friction}=\mu mg\cos\gamma$$

where $F_{friction}$ is the magnitude of the total frictional force on the robot, μ is the friction factor of the robot, its value is related to both the magnitude of the static friction and the magnitude of the sliding friction of the robot, the specific values and variation laws are given in Equation (24). m is the mass of the robot, $F_{gravity}$ is the component of the robot’s gravity along the slope direction, $F_{up}$ is the force that the robot needs to provide upwards along the slope during the uphill climb. Because the robot is subjected to frictional forces down the slope and the downward component of gravity along the slope, the robot must provide a corresponding upward force along the slope to ensure smooth robot operation.

Because it exists during the operation of the robot:

(19) $$F_{up}=F_{wheels}$$

So, the power consumption of the robot can be extended to:

(20) $$\begin{array}{ll} P_{motion} & =\lambda_{1}\sum_{i=1}^{4}\left(v_{i}*\frac{△v}{△t}\right)+\lambda_{2}\left(\sum_{i=1}^{4}\frac{△v_{i}}{△t}*v^{2}*t\right)+\lambda_{3}\left(t^{2}*\frac{△v}{△t}*v^{2}\right)+\mu^{2}k_{1}m^{2}g^{2}\cos^{2}\gamma \\ & +\mu \left(2mgk_{1}\sin\theta \sin\gamma +\omega k_{2}\right)mg\cos\gamma +mg\sin\gamma \sin\theta \left(mgk_{1}\sin\gamma \sin\theta +\omega k_{2}\right)\\ & +\mu_{1}\sum_{i=1}^{4}\left(N_{i}\cdot v_{i}\right)\cos\theta +\mu_{2}N\cdot v\cos\theta \cos\gamma +\tau \cdot {\left(F_{gravity}+F_{friction}\right)}^{2}+k\left(T\right)+\beta (T)*t \end{array}$$

#### 4.1.2. Power Analysis of Robot Downhill Process

During the descent, the frictional force of the robot is always upward in the direction of the slope; the component of gravity along the direction of the slope is smaller than the frictional force of the robot. In order to ensure that the robot can move smoothly downward, the robot needs to provide a drive force downward along the slope to ensure the balance of forces on the robot. The relationship between the forces at this point is:

(21) $$F_{down}=F_{friction}-F_{gravity}$$

$F_{down}$ is the upward force along the slope that the robot needs to provide during the descent, because the combined force formed by the component of gravity along the slope and the component of friction along the slope is downward along the slope. During the whole movement, the robot is force balanced, the robot’s wheels will roll down in the direction of the slope, and there will be no reverse rotation of the wheels. For Mecanum wheel robots, due to the special design of the Mecanum wheels (the rollers of the Mecanum wheels can rotate around themselves), when the component of gravity along the slope is bigger than the limit of sliding friction between the roller and the slope (i.e., the limit of rolling friction of the Mecanum wheels), the robot will lose the ability to control its own motion state (this out-of-control state we do not study). For ordinary vehicles, the limit of downhill is the sliding friction between the tire and the contact surface force limit. This limit is greater than the rolling friction limit of the drive wheel, Therefore, the process of $F_{down}$ increasing in the opposite direction will occur. For an omnidirectional wheel robot, such as the Mecanum wheel, there is a trend of $F_{down}$ decreasing to 0. So,

(22) $$\begin{array}{ll} P_{motion} & =-\lambda_{1}\sum_{i=1}^{4}\left(v_{i}*\frac{△v}{△t}\right)+\lambda_{2}\left(\sum_{i=1}^{4}\frac{△v_{i}}{△t}*v^{2}*t\right)+\lambda_{3}\left(t^{2}*\frac{△v}{△t}*v^{2}\right)+\mu^{2}k_{1}m^{2}g^{2}\cos^{2}\gamma \\ & -\mu \left(2mgk_{1}\sin\theta \sin\gamma +\omega k_{2}\right)mg\cos\gamma +mg\sin\gamma \sin\theta \left(mgk_{1}\sin\gamma \sin\theta +\omega k_{2}\right)\\ & +\mu_{1}\sum_{i=1}^{4}\left(N_{i}\cdot v_{i}\right)\cos\theta +\mu_{2}N\cdot v\cos\theta \cos\gamma +\tau \cdot {\left(F_{gravity}+F_{friction}\right)}^{2}+k\left(T\right)+\beta (T)*t \end{array}$$

### 4.2. The Influence of the Center of Gravity on the Power Consumption of the Robot

The offset of the robot’s center of gravity relative to the center position directly affects the iron consumption and mechanical output of the robot. The impact on iron consumption is mainly reflected in the impact on the friction force, i.e., on the positive pressure of each wheel, which is defined as F=μ  × N, where N is the positive pressure on the contact surface. For a robot whose center of gravity deviates from the center of gravity, the positive pressure on the contact surface of each of its wheels is different, where the positive pressure on the contact surface can be expressed as:

(23) $$N_{i}=\left(\frac{1}{2}\pm \frac{\delta_{a}}{a}\right)\left(\frac{1}{2}\pm \frac{\delta_{b}}{b}\right)\cdot N$$

where N is given by Equation N=mgcosγ; m, g, γ represent the mass of the robot, the gravity constant, and the inclination angle of the terrain; a and b represent the horizontal and vertical distances between each wheel; $\pm \delta_{a}$ and $\pm \delta_{b}$ represent the distance between the center of gravity of the robot and the center of the robot [2].

**Figure 3:**
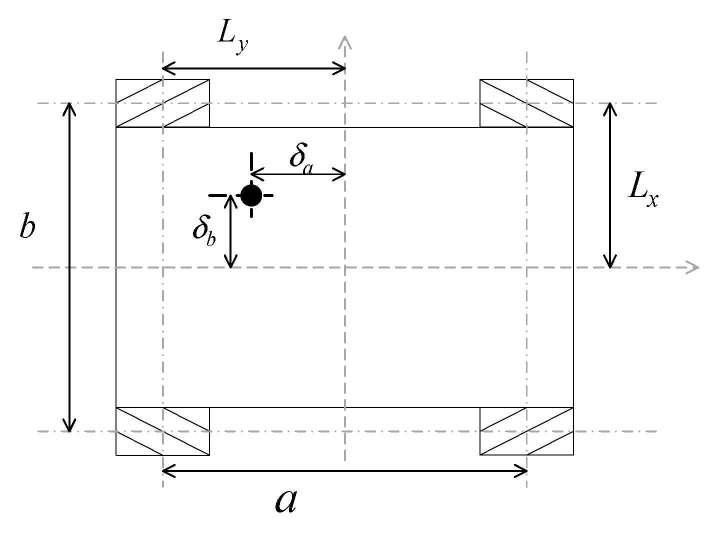
Schematic diagram of the analysis of the robot’s center of gravity position.

Figure 3 shows a diagram of the change in the robot’s center of gravity, with the black dots indicating the current position of the robot’s center of gravity. The $\delta_{a}$ and $\delta_{b}$ indicate the lateral and vertical shift of the robot’s center of gravity.

(24) $$\mu =\frac{R_{0}+\delta_{a}}{R_{0}+L_{y}}\cdot \mu_{R}+\frac{2a\left(\frac{1}{2}-{\left(\frac{\delta_{b}}{a}\right)}^{2}\right)}{R_{0}+L_{x}}\cdot \mu_{S}$$

(25) $$\mu_{1}=\frac{L_{x}+\delta_{b}}{R_{0}+b}\cdot \mu_{R}+\frac{L_{y}+\delta_{a}}{R_{0}+a}\cdot \mu_{S}$$

(26) $$\mu_{2}=\frac{L_{x}-\delta_{a}}{R_{0}+L_{x}}\cdot \mu_{R}+\frac{L_{y}-\delta_{b}}{R_{0}+L_{y}}\cdot \mu_{S}$$

$\mu_{R}$ and $\mu_{S}$ correspond to the sliding friction and rolling friction of the robot, $R_{0}$ corresponds to the turning radius of the robot, for the Mecanum wheel robot, because of its special construction, the friction factor is different when moving with different turning radius. $L_{x}$ and $L_{y}$ are the distances from the center of the driving wheel to the center of the vehicle, as shown in Figure 2.

### 4.3. The Effect of Temperature on the Power Consumption of the Robot

The energy consumption of three systems of the robot is temperature related. As the robot moves, part of the energy of the motor will be converted into heat to be lost. As the temperature of the motor increases, the torque and speed constant of the robot will decrease [38]. To compensate for the decreased torque, it is necessary to increase the current input to the motor. Since both $I_{a}$ and $R_{a}$ in Formula (3) increase, the temperature of the robot will continue to rise. Therefore, if the heat cannot be dissipated reasonably, the robot will lose more and more energy. At the same time, the increased current flowing through the motor drive board will cause the energy consumed by the motor drive board to increase accordingly, and the resulting speed change will cause the control system to lose more energy.

The change of resistance with temperature conforms to the formula:

(27) $$R\left(T\right)=R_{0}\left(1+\alpha \left(T-T_{0}\right)\right)$$

Among them, $R\left(T\right)$ represents the resistance value of the robot when the temperature is T, α is the temperature coefficient, T represents the temperature at the current time, $R_{0}$ and $T_{0}$ represent the resistance value at a certain time and the temperature at this time.

Table 1 shows the parameters of the robot motors, which are only related to the type of motor used in the robot and are not related to the robot structure.

(28) $$k_{1}\left(T\right)=k_{10}{\left(1\;+\;\alpha_{R}\left(T\;-\;T_{0}\right)\right)}^{-2}{\left(1\;+\;\alpha_{M}\left(V-V_{max}\right)\right)}^{-4}$$

(29) $$k_{2}\left(T\right)=k_{20}\left(1\;+\;\alpha_{R}\left(T\;-\;T_{0}\right)\right){\left(1\;+\;\alpha_{M}\left(V+V_{max}\right)\right)}^{-2}$$

(30) $$k\left(T\right)=k_{10}k_{20}{\left(1\;+\;\alpha_{R}\left(T\;-\;T_{\;0}\;\right)\right)}^{-2}{\left(1\;+\;\alpha_{M}\left(V+V_{max}\right)\right)}^{-4}$$

(31) $$\beta \left(T\right)=\;k_{20}{\left(1\;+\;\alpha_{M}\left(V+V_{max}\right)\right)}^{-2}$$

where $k_{10}$ and $k_{20}$ are the motor constants measured at the temperature of the motor, $T_{0}$ is the temperature coefficient of the motor, $\alpha_{R}$ and $\alpha_{M}$ is the speed coefficient of the motor.

Table 2 shows the temperature parameters of the robot motors, which are only related to the type of motor used in the robot.

## 5. Simulation and Experimental Verification

The experimental setting is mainly to study the power consumption of the robot in various situations during uphill and downhill. Before the experiment, we set up a test bench that can freely adjust the uphill and downhill angles. In order to ensure that the power of the robot will be stable on the test bench, we set the slope of the test bench to 4 m during the test. The robot we used in the experiment is a four-wheeled Mecanum wheel robot called MiniBalance, in our laboratory. The main parameters of the robot are the mass of the robot: m=12 kg, the overall dimensions of the robot are length: $L_{1}=0.86\;m$, width: $L_{2}=0.52\;m$, the motor of the robot is the planetary gear motor model MD36N, the design of the robot is independent suspension, which has good shock absorption function and can ensure that the robot can still be used normally in the process of shifting the center of gravity, the maximum load weight of the robot is 30 kg. The robot is designed with 24 v power supply and the control board of the bottom control system is stmf407.

Table 3 shows the parameters of the theoretical model established above. By substituting the parameters into the theoretical model established above and implementing the model mathematically in MATLAB, we can obtain the simulated power consumption values in this case.

During the experiment, we increased the height of the test bed continuously, and used inclinometer (the accuracy of the inclinometer can reach $0.05^{\circ }$) for accurate angle measurement, then control the FMWR to start from the flat bottom first, and then go uphill when the speed is stable. This process requires PID to intervene to ensure that the speed of the robot remains constant during the uphill. When the robot stabilizes in the uphill process, it immediately enters the horizontal movement stage to buffer, and then enters the downhill stage. During the downhill process, the robot control system needs to control the speed of the robot to travel at a preset speed to prevent out of control situations. In order to study the influence of various factors in the environment on the power consumption of the robot, we use different speeds and temperatures and the center of gravity to perform the same experimental process during the experiment. We verify the correctness of our model by comparing the differences in power consumption.

**Figure 4:**
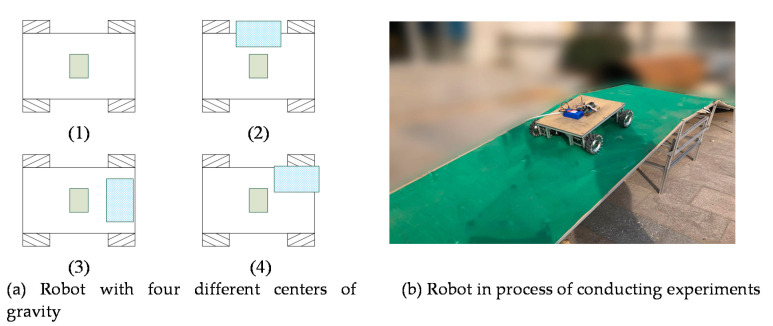
Robot experiment process diagram.

Figure 4 shows the experimental pictures of the robot in the real scene. Figure 4a shows a schematic diagram of the robot’s center of gravity by changing the position of the weight loaded on the robot. The blue object in the figure is iron weighing 10 kg. Figure 4b shows the robot during the uphill and downhill experiments. From the picture, we can see that we use a green rubber carpet to increase the friction, and the angle of the uphill and downhill can be adjusted freely.

**Figure 5:**
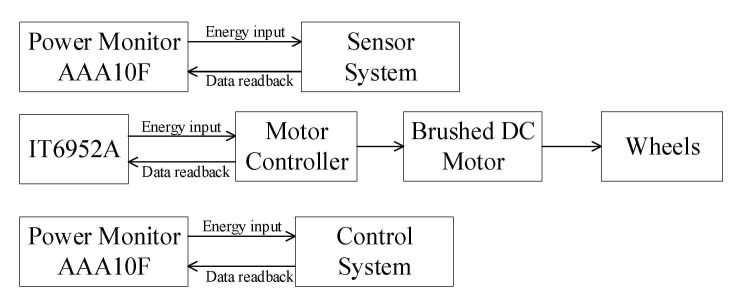
Power measurement system.

The measurement method adopted by the robot is shown in Figure 5. For the control system and the sensor system, the power is less than 10 W and the sampling value of the power consumption is relatively high. We use power monitor AAA10F to measure power consumption. For the motion system, because of its high power, we use IT6952A to measure. The actual measurements shown in the article are averaged after several experimental measurements, and we operate in accordance with statistical principles. This ensures that the results of the experiments are not subject to chance and guarantees the accuracy of the experimental validation as well as the reproducibility of the experiments.

**Figure 6:**
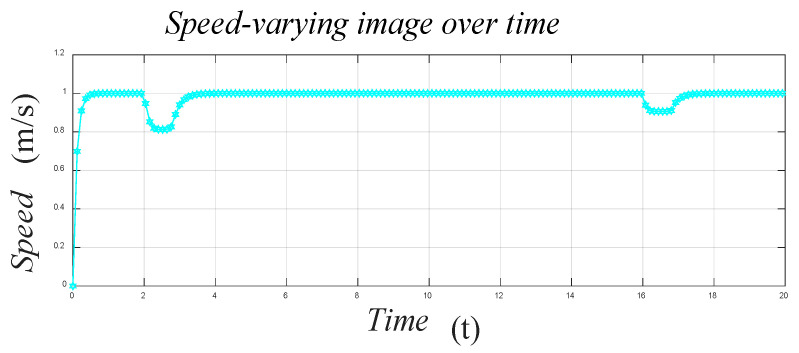
The speed of the robot during the movement.

Before the experimental verification of each system, first, the speed variation during the motion should be analyzed and modeled. Figure 6 shows the speed variation of the robot throughout its motion when we set the maximum speed of the robot to 1 m/s.

### 5.1. Motion System

**Figure 7:**
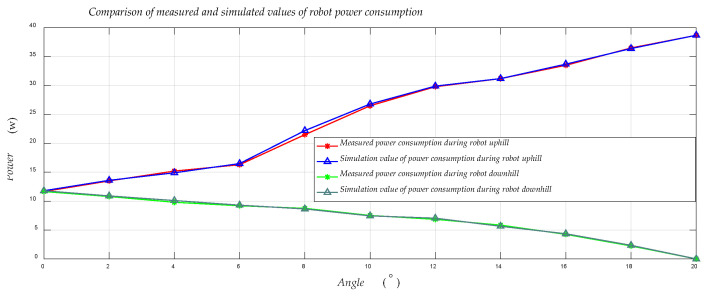
Comparison between the measured value and the theoretical value when the power consumption is stable during the robot’s uphill and downhill.

**Figure 8:**
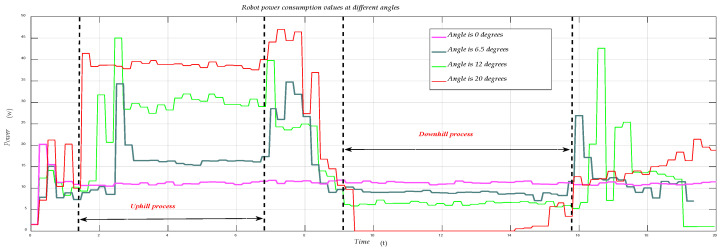
Power consumption during robot movement.

For the motion system, we need to verify that the power consumption of the robot fluctuates due to changes in slope and center of gravity during the robot’s movement. The first is the change in the power consumption of the robot’s control system due to changes in the angle of the slope and the speed of the robot itself during the uphill process:

Figure 7 shows the comparison between the measured and simulated values of the robot’s power during the process of uphill and downhill. In the validation process, with two degrees as a sampling interval, we performed experimental validation and MATLAB simulation, and compared the solved simulated values with the experimental measured values, and it can be seen through Figure 7 that the simulated values are very close to the actual measured values. The accuracy of our modeling is proved to be relatively high. Figure 8 shows the real-time power consumption of the robot during its entire movement.

Through Figure 8, we can see that the robot’s power has a very significant rise in the startup process, the process of starting uphill, and the process of finally entering the flat. This is because, in these several processes, the speed of the robot will decrease due to the power being unable to increase instantly. Because of the role of PID control, the robot will increase its power to increase the speed to a predetermined value, so the process of increasing the power consumption of the robot corresponds to the process of increasing the speed of the robot.

As the robot’s uphill angle increases, the power difference between the robot’s uphill and downhill will increase; according to our research, the Mecanum wheel robot reached the limit of the robot’s uphill and downhill at 20 degrees due to its special structure. At 20 degrees, it is both the limit for the robot to go uphill and the limit for the robot to go down. During the descent, the robot has a long-term power of 0.

**Figure 9:**
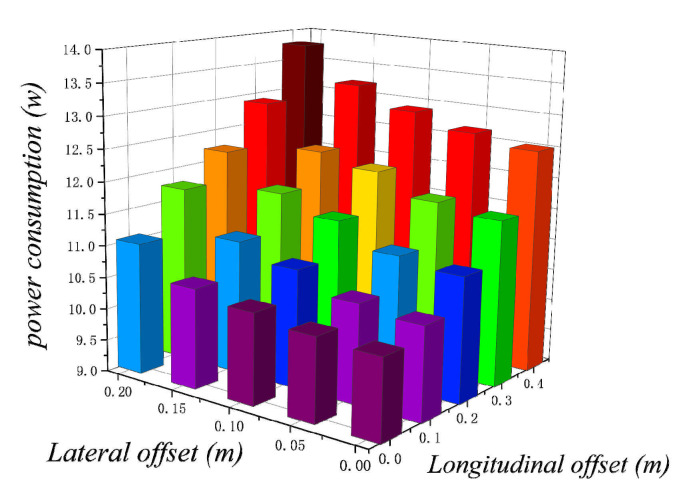
Influence of the shift of the center of gravity on the power consumption of the robot.

Figure 9 shows the effect of the robot’s center of gravity offset on the position of the robot’s center of gravity on the power consumption of the robot. The data in Figure 9 are the actual measurements of the effect of the change in the robot’s center of gravity on energy consumption. In order to show the results more concisely, we only show the actual measured values here, and the error between the simulated and actual values does not exceed 0.5 w after the actual comparison.

For the shift of the center of gravity, the method shown in Figure 4a is adopted; the position of the center of gravity of the robot is shifted by placing heavy objects at different positions of the robot. Figure 9 shows the position of the center of gravity of the robot gradually shifted outward from the position of the center of the robot; through Figure 9, it can be seen very clearly that the power consumption of the robot increases significantly as the center of gravity of the robot shifts outward. As the center of gravity of the robot shifts outward, the load on one or more wheels of the robot will increase significantly, which will lead to the increase of power consumption of the corresponding motor of the robot. Moreover, the increase of power consumption is not a linear relationship with the movement of the robot’s center of gravity, but a feature of exponential increase—that is, the farther the robot’s center of gravity is from the center, the more energy consumption is increased for the same outward movement of the robot’s center of gravity. This kind of shift of the center of gravity is very likely to cause the overload situation of the robot motor.

**Figure 10:**
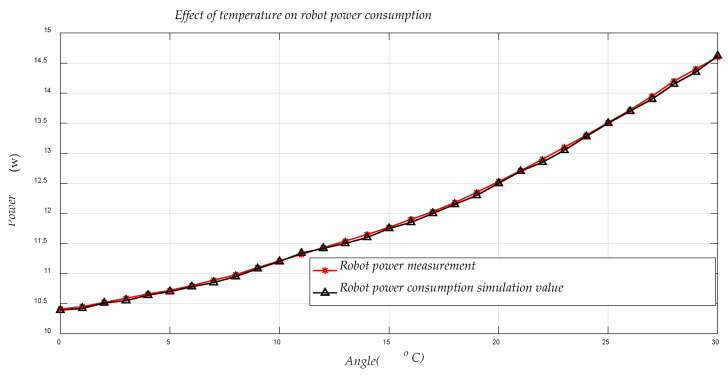
Influence of temperature on the power consumption of robot motion system.

Figure 10 shows the variation of the energy consumption of the robot’s motion system with the change of temperature, which has a very obvious effect on the energy consumption of the motion system. It is important to note that the data shown in Figure 9 and Figure 10 are the instantaneous power values collected after the robot changes temperature or the center of gravity and reaches a steady state of operation, and each of the data here is obtained by averaging the data from multiple experiments, so that the effects of accidental factors can be excluded. The higher the temperature of the robot, the higher the energy consumption of the motion system.

### 5.2. Control System

The verification of the robot control system’s power consumption is mainly to verify the fluctuation of the power consumption of the robot’s control system during the process of uphill, downhill, and final stabilization of the robot. Test the accuracy of our power consumption modeling of the robot control system.

**Figure 11:**
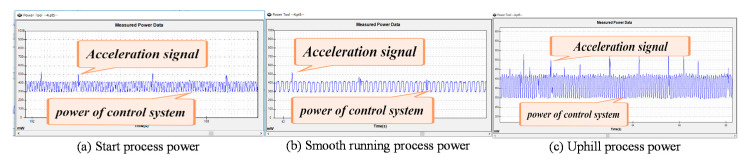
Power of the control system in different states of motion.

Figure 11 shows the power consumption of the control system at different stages of the robot’s movement. Through Figure 8, we can clearly see that the basic consumption of the robot control system is basically the same. The difference is the amount of data processed by the robot at different stages and the acceleration pulses issued by the robot due to different speeds. According to our theory, the robot has the largest number of acceleration pulses during the uphill process, followed by the startup process, and finally the process of stable operation. The above figure is very good to confirm our idea, so it can explain our modeling is accurate.

### 5.3. Sensor System

**Figure 12:**
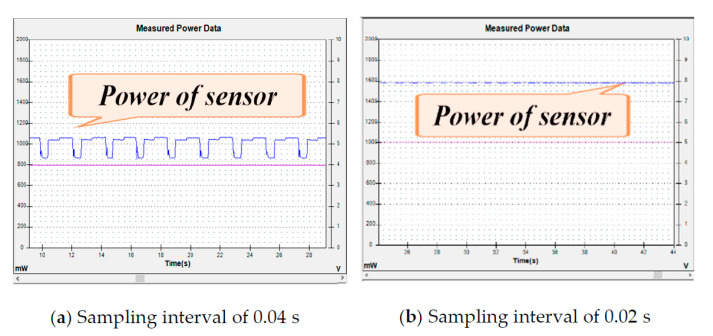
Power of the sensor system.

The power consumption of the sensor system is closely related to the speed of the robot. When the speed of the robot is not very fast, the sensor can operate in a non-full-scale way. When the speed of the robot increases gradually, the frequency of the sensor gets higher and higher, so the power generated will be higher and higher (see Figure 12).

Figure 12 shows the robot sensing system’s power consumption at different sampling frequencies. Figure 12a shows the energy consumption of the sensing system when the robot sampling frequency is 0.04 s. Figure 12b shows the energy consumption of the sensing system when the robot sampling frequency is 0.02 s. According to the graph, it can be concluded that the higher the sampling frequency of the sensor, the higher the energy consumption of the sensing system. According to the setting, the faster the speed of the robot, the higher the sampling frequency will be, and by generalizing it, we can get that the faster the speed of the robot, the higher the energy consumption of the sensing system. We demonstrated the energy consumption characteristics of the sensor system in the previous research article [24,25], so we will not elaborate on them here.

## 6. Discussion and Conclusions

Before comparing the robot energy consumption modeling with the actual energy consumption measurements, we first measure the energy consumption of the robot during motion several times and find out the actual power value of the robot during motion, according to the statistical processing method.

**Figure 13:**
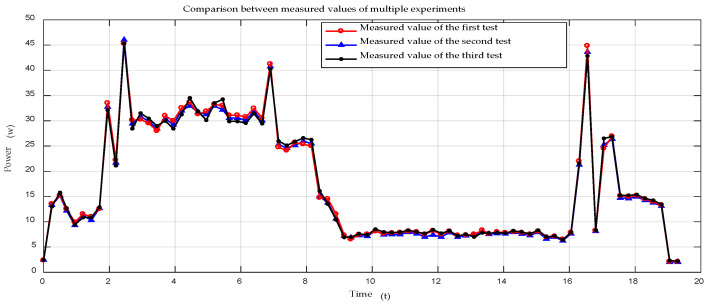
Comparison of power consumption values from multiple experimental measurements.

**Figure 14:**
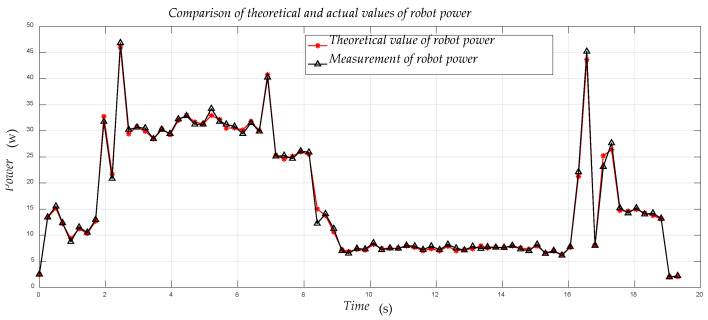
Comparison of the measured value of the robot’s real-time power with the actual value during the movement.

Figure 13 shows the comparison of multiple experimental measurements of the robot’s energy consumption throughout the motion of the robot. The comparison shows that the results of each experimental measurement are very close to each other and are consistent with the results of the robot’s energy consumption measurements shown in Figure 13, which shows that our experimental results are not influenced by chance. The data in Figure 14 are averaged over several experimental measurements, which ensure the accuracy of the robot modeling verification.

Figure 14 shows the relationship between the actual and measured values of the robot’s power during the entire movement of the robot. The error between the theoretical and actual values of the calculated power of the robot is within 5%, and the accuracy rate is about 95%. It can be proved that the modeling of the three major robot systems is very successful.

By comparing the modeling with actual values, it is found that the accuracy of the robot modeling is about 95%, and the modeling has reached the expected modeling accuracy. The difference between the modeling and the actual value is mainly because the consumption of the robot’s other parts of its motion system during the modeling process has not been thoroughly searched.

This paper proposes a power consumption model of a robot in a complex environment. The influences of the angle of the upslope and downslope of the robot during the movement, the temperature of the robot, and the change of the center of gravity of the robot on the power consumption of the robot are fully studied. Compared to traditional modeling, we pay more attention to the fluctuation of the power consumption of the robot during the transition of the different motion states of the robot. By incorporating the fluctuations of the robot into the modeling, we improved the accuracy of the robot modeling to about 95%, which greatly improved the accuracy of the robot modeling. At the same time, we analyzed the state of Mecanum wheels during actual operation and found that Mecanum wheel robots and ordinary wheeled robots are different in the process of uphill and downhill. The angle limit of the Mecanum wheel robot on uphill and downhill is much smaller than that of the ordinary wheeled robot. This is due to the particular structure of Mecanum wheels—that is, the Mecanum wheel robot sacrifices the robot’s ability to adapt to complex environments, to increase the flexibility of the robot.

## Figures and Tables

**Table 1:** Friction constants as measured through different wheels.

	Front	Back	Mean
M1 (Left motor)			
$k_{10}$	0.0019	0.0021	0.0020
$k_{20}$	1.5223	1.5325	1.5274
M2 (Left motor)			
$k_{10}$	0.0022	0.0026	0.0024
$k_{20}$	1.5445	1.5326	1.5385

**Table 2:** Friction constants as measured through different wheels.

	Front	Back	Mean
M1 (Left motor)			
$\alpha_{R}$	1.25	1.56	1.405
$\alpha_{M}$	1.32	1.44	1.38
M2 (Left motor)			
$\alpha_{R}$	1.35	1.37	1.36
$\alpha_{M}$	1.38	1.35	1.365

**Table 3:** The value of parameter in the theoretical model.

Parameter	Value
$\lambda_{1}$	0.15
$\lambda_{1}$	0.08
$\lambda_{1}$	0.01
$\rho_{1}$	0.2
$\rho_{2}$	0.05
τ	0.3
$T_{0}$	20
